# Ophthalmia Neonatorum

**Published:** 1890-01-11

**Authors:** 


### The Practitioner's Outlook and Retrospect.

*[The Editor will be glad to receive offers of co-operation and contributions from members of the profession. There is no doubt that much valuable material full of interest and instruction is at present lost to science. This is largely so in the case of (1) the younger and less-known members of the hospital staff, (2) those connected with cottage hospitals, and (3) the great body of medical practitioners not attached to any institution. The co-operation of all is cordially invited to enable the Editor to make* The Hospital *of the utmost possible use and value to the profession. Specialists willing to review books on their special subjects are invited to communicate this fact at once. All letters should be addressed to* The Editor, The Lodge, Porchester Square, London, W.]

### MEDICINE.

### OPHTHALMIA NEONATORUM.

As to the true cause of this very common affection, there is no longer any difference of opinion, and astringent lotions have given place to germicidal solutions, the practical question being to find one sufficiently powerful to destroy the gonococcus, yet not so as to injure the delicate structure of the infant's eye. That hitherto in favour in most continental foundling and lying-in hospitals has been a two per cent. nitrate of silver. Velorer, indeed, considers a one per cent. solution too weak; but Levow maintains that it is only necessary to use a little more, three or four drops instead of two, to obtain equallv good results. Dr. Pomeroy, of New York, reports a case in which two drops of a two per cent. solution caused such hæmorrhage from the conjunctiva as could be arrested only by a firm compress, secured by an elastic band. But while the best success has attended assiduous washing of the eyes with pure water, or such innocent antiseptics as a 3 per 1,000 solution of salicylic acid, the general feeling in Germany and Russia, especially in Vienna, seems to be in favour of 1 per 1,000 solution of sublimate, or of beginning in every case with a much weaker solution as a prophylactic, and employing the stronger as a cure when any symptoms of ophthalmia make their appearance.

